# Organic vegetable juice supplement alleviates hyperlipidemia in diet‐induced obese mice and modulates microbial community in continuous colon simulation system

**DOI:** 10.1002/fsn3.3193

**Published:** 2023-01-13

**Authors:** Hyeon Ji Kim, Sung Joon Mo, Jisoo Kim, Bora Nam, Soo‐Dong Park, Jae‐Jung Sim, Jaehun Sim, Jung‐Lyoul Lee

**Affiliations:** ^1^ R&BD Center, hy Co., Ltd. Yongin‐si Korea

**Keywords:** colon simulation system, hyperlipidemia, lipid profiles, organic vegetable juice

## Abstract

In this study, we investigated the effects of organic vegetable juice (OVJ) supplementation on modulating the microbial community, and how its consumption ameliorated blood‐lipid profiles in diet‐induced obese mice. Here, we studied the alleviating effect of hyperlipidemia via animal experiments using diet‐induced obese mice and analyzed the effect of OVJ on the microbial community in continuous colon simulation system. OVJ consumption did not have a significant effect on weight loss but helped reduce the weight of the epididymis fat tissue and adipocytes. Additionally, blood‐lipid profiles, such as triglyceride, high‐density lipoprotein, and glucose, were improved in the OVJ‐fed group. Expression levels of genes related to lipid synthesis, including SREBP‐1, PPARγ, C/EBPα, and FAS, were significantly decreased. In addition, OVJ treatment significantly reduced inflammatory cytokines and oxidative stress. OVJ supplement influenced intestinal bacterial composition from phylum to genus level, including decreased Proteobacteria in the ascending colon in the phylum. At the family level, *Akkermansia*, which are associated with obesity, were significantly augmented in the transverse colon and descending colon compared to the control juice group. In addition, treatment with OVJ affected predicted lipid‐metabolism‐function genes related to lipid synthesis. These results suggest that OVJ supplementation may modulate gut microbial community and reduce the potential symptom of hyperlipidemia in diet‐obese mice.

## Graphical abstract

The antihyperlipidemic effect of organic vegetable juice (OVJ) was studied through animal experiments using diet‐induced obese mice, and the effect of OVJ treatment on the microbiome was analyzed in a continuous colon simulation system. Supplementation of OVJ may alleviate hyperlipidemia by improving blood lipid profile and antioxidant effects in obese mice, and alter the microbiome to affect predicted lipid metabolism function genes involved in lipid synthesis. These results suggest that OVJ supplementation may modulate the gut microbiome and reduce the potential role of hyperlipidemia.

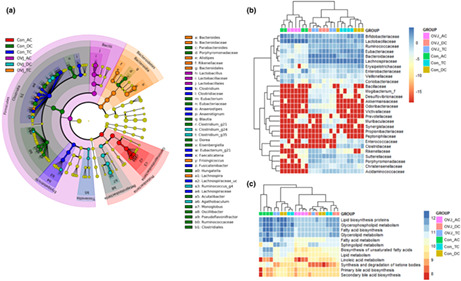

## 1 INTRODUCTION

Vegetables are a major source of phytochemicals with potential beneficial health properties (Nuutila et al., 2003; Singh et al., 2009). Among the major phytochemicals of vegetables are polyphenols and carotenoids. Polyphenols have been studied for their antioxidant properties of preventing the damage caused by reactive oxygen species (ROS), such as hydroxyl radicals, hydrogen peroxide (H_2_O_2_), and superoxide (Williams et al., 2013). Carotenoid compounds have been studied for their anti‐obesity and anti‐inflammatory effects (González‐Castejón & Rodriguez‐Casado, 2011). Intake of vegetables prevented damage by oxidative stress and improved hyperlipidemia according to previous studies. The relationship between lipid levels in the blood and the level of antioxidants from vegetables has been recently suggested (Kim et al., 2008; Yang et al., 2008).

Obesity occurs because of many factors, such as energy imbalance, neurosecretion factors, and environmental factors. Recently, the number of obese patients has sharply increased due to westernized diet, stress, and lack of exercise, and obesity has been a major health problem worldwide (Jeung et al., 2019). Obesity causes various diseases, such as metabolic syndrome, cholelithiasis, cardiovascular disease, hypertension, diabetes, and hyperlipidemia (Bray, 2000). Hyperlipidemia refers to a condition that causes inflammation due to the presence of more fatty substances than necessary in the blood (Williams et al., 2013). Although hyperlipidemia does not have any specific symptoms, it is a risk factor for high blood pressure, arteriosclerosis, and stroke, and significantly increases the mortality rate in cardiovascular diseases (Klop et al., 2013; Nelson, 2013).

The human gut microbiota is composed of more than 1 million bacteria and a complex community of over 100 trillion diverse bacteria (Doré & Blottière, 2015; Graf et al., 2015). The gastrointestinal tract (GIT) includes a diverse and complex microorganism community, which are major contributors to human health (Mangal et al., 2017). The human digestive system has no digestive enzymes for plant‐derived complex carbohydrates, but the human gut microbiota can decompose and utilize them (Graf et al., 2015). In addition, gut microbiota plays a role in producing organic acid and short‐chain fatty acids (SCFAs) including propionate, butyrate, and acetate in the human GIT. These SCFAs are known to affect and regulate the microbial composition of the human intestinal microbiota (Den Besten et al., 2013). For these reasons, studies of human intestinal microbiome using genomic and metagenomic analyses on various topics have been actively conducted in recent years.

The in vivo analysis of human or animal gut is an ideal method to investigate intestinal microbiota, but these methods have technical and ethical difficulties, including expensive experimental cost, long time consumption, and difficulty in standardization due to limitations in controlling individual diets (Cha et al., 2018). In addition, in vivo studies are limited to fecal samples, which do not provide information about dynamic microbial changes in the fermentation site of the gut (Sousa et al., 2008). Furthermore, the gut microbiome of in vivo is often impaired due to inter‐individual differences associated with numerous factors such as age, gender, diet, geography, genetic background, and antibiotic use (Williams et al., 2015). To resolve these problems, an in vitro gut fermentation model has been developed and characterized by applying simple batch culture conditions and using a more complex apparatus for human fecal samples to control pH, temperature, and anaerobic conditions. A simple culture system, such as the TNO in vitro model, replicates the proximal colon in a single‐segment fermenter, whereas the three‐stage continuous system replicates the whole large intestine (Cinquin et al., 2006; Feria‐Gervasio et al., 2014).

Batch colon simulation models are generally closed systems, and these models are used with sealed vessels containing suspensions of fecal samples and culture system medium under anaerobic conditions. Batch culture systems have the advantage of being easy to set up and useful for fermentation, but these systems create a short time frame for fermentation research and are unable to control microorganisms. In contrast, continuous culture systems are open systems, where the fresh medium is injected and waste is released periodically. These systems simulate the major parts of the large intestine, including the ascending colon (AC), transverse colon (TC), and descending colon (DC). Continuous colon simulation systems are well‐controlled environmental parameters, which enable the detection of changes in metabolites and microbial composition in each part of the large intestine (Adamberg et al., 2014; Costabile et al., 2015; Maccaferri et al., 2012). Therefore, continuous colon simulation systems are more similar to the human GIT compared to the batch model.

This study investigated whether organic vegetable juice (OVJ) can modulate large intestinal microbiota and affect the predicted lipid metabolism function genes using continuous colon simulation systems. Based on the results of continuous simulation systems, we studied the effects of OVJ on the blood‐lipid levels, liver gene expression, and adipocytes in the epididymal fat of diet‐induced obese mice. In addition, we investigated the effects of OVJ on the antioxidant levels of diet‐induced obese mice.

## 2 MATERIALS AND METHODS

### 2.1 Animal experiments

**FIGURE 1:**
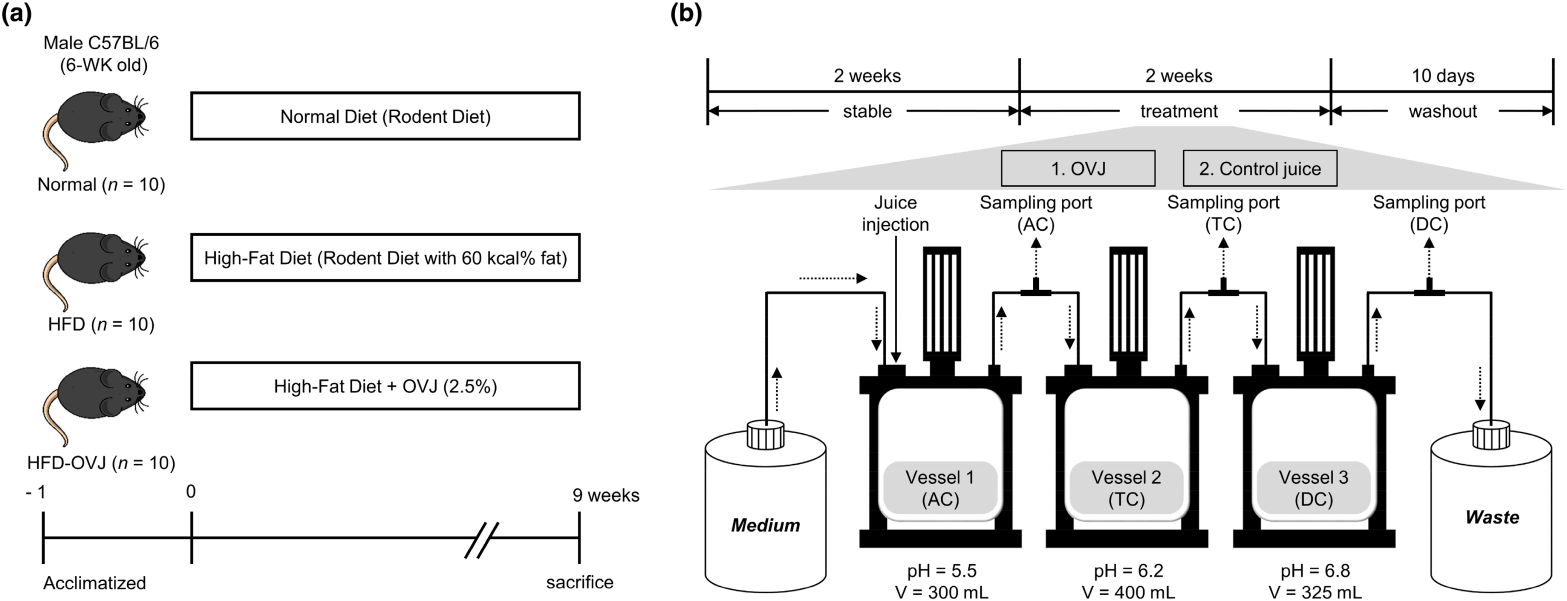
Schematic of experimental design. (a) Six‐week‐old male C57BL/6 mice were randomly assigned to either Normal (*n* = 10), HFD (*n* = 10), and HFD‐OVJ (*n* = 10) groups. Each group were fed the following diet for 9 weeks: Normal group, rodent diet; HFD group, HFD (rodent diet with 60 kcal% fat); HFD‐OVJ group, HFD with 2.5% OVJ. (b) Schematic representation of the continuous colon simulation system. AC, ascending colon; DC, descending colon; HFD, high‐fat diet; HFD‐OVJ, organic vegetable juice with high‐fat diet; TC, transverse colon

Figure 1a shows the design of the in vivo experiments performed in this study. Six‐week‐old male C57BL/6 was purchased from Laonbio. All mice were fed at constant humidity (55 ± 10%) and temperature (22 ± 1°C) with a 12 h light/dark cycle. After 7 days of acclimatization, mice were fed a normal diet group (*n* = 10; AIN‐93G), high‐fat diet (HFD) group (*n* = 10; Rodent diet with 60 Kcal% fat), and high‐fat with organic vegetable juice (HFD‐OVJ) (*n* = 10, group) for 9 weeks. The composition of the ND was formulated based on the AIN‐93G purified rodent diet. In this study, the recommended daily intake of OVJ obtained from hy Co., Ltd. was applied for mice to examine the health effects of OVJ. Table S1 shows the configuration of the OVJ. The OVJ was freeze‐dried, evenly mixed with a HFD in powder form, and then supplied to mice in the HFD‐OVJ group. Body weight and food intake were measured weekly. The food efficiency ratio (FER) was calculated by applying the equation: 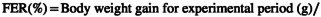 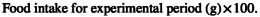 Blood samples were taken from the inferior vena cava and immediately placed at room temperature (20–23°C) and then centrifuged at 3000 *g* at 4°C for 10 min. The serum was separated from the blood sample. The harvested serum was stored at −80°C until analysis. The liver and epididymis fat tissues were collected, rinsed with sterilized PBS, and weighed. The partial liver tissue was stored in a deep freezer at −80°C immediately after collection for gene expression analysis using real‐time PCR. The animal experimental plan was approved by the Ethics Committee at the R&D Center, hy Co., Ltd. (AEC‐2020‐00003‐Y).

### 2.2 Blood biochemistry analysis

Serum samples were collected at T&P Bio for blood analysis. The serum total cholesterol (T‐CHOL), triglyceride (TG), high‐density lipoprotein cholesterol (HDL), low‐density lipoprotein cholesterol (LDL), glucose (GLU), aspartate transaminase (AST), and alanine transaminase (ALT) levels were determined using Beckman Coulter AU480 analyzer (Beckman Coulter Inc.). Serum samples remaining after the blood biochemistry tests were used to measure the following antioxidant biomarkers: (a) 8‐hydroxy‐2‐deoxyguanosine (8‐OHdG) ELISA Kit (Abcam) was used. (b) malondialdehyde (MDA) concentration in serum samples was measured using a lipid peroxidation Assay Kit (Abcam). (c) H2O2 level was measured using a Catalase Assay Kit (Colorimetric/Fluorometer) (Abcam).

### 2.3 Histological tissue analysis

The liver and epididymis fat tissues were washed with sterilized PBS and fixed in 10% formalin. Tissue samples were obtained from T&P Bio for histological analysis. Fixed tissues were implanted in paraffin for hematoxylin and eosin staining. The liver and epididymis fat tissues were observed under a fluorescence microscope (Axiovert 200M, Carl Zeiss) at a magnification of ×20 and ×10, respectively.

### 2.4 RNA extraction and gene expression analysis

Total tissue RNA was extracted from liver tissues using the easy spin Total RNA Extraction Kit (iNtRON) via bead‐beating. The liver tissues were mixed with 1 ml lysis buffer and transferred into lysing matrix tubes, containing specialized beads (MP Biomedicals), and pulverized through Fastprep24. After bead‐beating, the remaining procedure of easy spin Total RNA Extraction Kit was followed. Consequently, total RNA was eluted with 50 μl elution buffer. Total RNA samples were quantified using Nanodrop and stored at 20°C until gene expression analysis. The extracted total RNA was reverse‐transcribed into cDNA using an Omniscript RT Kit (Qiagen). Reverse transcription PCR conditions were set at 37°C for 60 min. The cDNA was amplified using a QuantStudio 6 Flex‐Real Time Instrument with a gene expression master mix. Real‐time PCR was performed using mouse‐specific TaqMan Gene Expression Assays and normalized by the expression of GAPDH (Applied Biosystems). Table S2 shows the catalog numbers of the genes and names.

### 2.5 The continuous colon simulation system

Figure 1b shows the workflow of the continuous colon simulation system performed in this study. Fecal samples were collected from three healthy adults who had not taken antibiotics for 3 months. Fecal samples were diluted with phosphate‐buffered saline (PBS) at a 1:10 (v/v) ratio in an anaerobic chamber. The fecal slurry sample was separated into each vessel at a final concentration of 2%. The continuous colon simulation system medium contained 1 g/L peptone, 4 g/L mucin, 0.5 g/L l‐cystein‐HCl, 1 g/L xylan, 0.5 g/L inulin, 0.4 g/L bile salt, 0.0025 g/L resazurin, 3 g/L yeast extract, 0.4 g/L d‐glucose, 2 g/L pectin, 1 g/L arabinogalactan, and 3 g/L starch. To simulate the environment of the large intestine, conditions were created for the three major parts of the large intestine; AC, TC, and DC. The volume and pH were as follows: 300 ml, pH 5.5 for AC; 400 ml, pH 6.2 for TC; 325 ml, pH 6.8 for DC (Cha et al., 2018). To maintain the anaerobic conditions in the simulation system, nitrogen gas (N_2_) was constantly injected at a flow rate of 10 ml/min. The temperature of each vessel was maintained at 37°C and the pH was automatically adjusted with 1 N HCl and 1 N NaOH. In addition, the pump on/off time interval was controlled to 12.5 ml/h to maintain a continuous flow. The colon system was pre‐run to achieve chemical and microbial stabilization for 2 weeks. After the stable step, the sample was treated in the AC and the washout step proceeded for 2 weeks.

### 2.6 Juice treatment and fecal slurry sampling from the culture system

Organic vegetable juice was obtained from the hy Co., Ltd. Control juice was diluted with water and mixed carbohydrates (glucose 4.44 g/200 ml, fructose 4.52 g/200 ml, and sucrose 7.84 g/200 ml). The amount of these monosaccharides and disaccharides was the same as the OVJ. Two hundred microliters OVJ and control juice were injected into the AC of the continuous colon simulation system per day for 2 weeks. The same amount of sample of fecal slurry from the colon simulation system was carried out at a constant time point at the same amount in each vessel. Sampling was performed a total of 8 times, once a day from 2 days before the end of the stable period, 4 days before the end of the juice treatment period, and 2 days before the end of the washout period. These fecal slurries were used to analyze microbial community.

### 2.7 Bacterial 16S rRNA amplification for next‐generation sequencing

The bioinformatics analysis of fecal DNA samples from the colon simulation system was carried out at Chunlab. Total fecal DNA samples were extracted using the QIAamp DNA Stool Mini Kit (Qiagen). PCR amplification of 16S rRNA sequences was conducted to prepare DNA sequencing templates. The V3–V4 region of the 16S rRNA sequence was amplified using the 341‐forward (341F)/805‐reverse (805R) primer set (341F: 5′‐TCGTCGGCAGCGTCAGATGTGTATAAGAGACAGCCTACGGGNGGCWGCAG‐3′; 805R: 5′‐GTCTCGTGGGCTCGGAGATGTGTATAAGAGACAGGACTACHVGGGTATCTAATCC‐3′). PCR conditions for the 341F/805R primer set were as follows: initial denaturation at 95°C for 3 min; 25 cycles of denaturation at 95°C for 30 s; annealing at 55°C for 30 s; extension at 72°C for 30 s; final elongation at 72°C for 5 min. After amplification of the 16S rRNA gene, the 16S rRNA amplicon library was used as a template DNA for next‐generation sequencing (NGS). Illumina MiSeq sequencing platform (Illumina) was used for NGS.

### 2.8 Bioinformatics analysis

The microbiome taxonomic profiling was analyzed using the EzBioCloud database provided by Chunlab. The raw data were analyzed using the QIIME 1.9.1 package program. The sequencing data were filtered for low‐quality reads and mismatched indexes using “Trim sequence” during quality control. High‐quality sequences of 300 bp were extracted using open reference clustering, one of several operational taxonomic unit clustering (OTU clustering) methods. After OTU clustering, Chimera checking was performed using ChimerSlayer. β‐diversity analysis was performed using unweighted UniFrac distances and visualized based on unweighted PCoAs. Linear discriminant analysis effect size (LEfSe) analysis (Segata et al., 2011) and PICRUSt platform were used for metagenome prediction (Langille et al., 2013). The data were analyzed using R version 3.6.2 (https://www.r‐project.org). All datasets have been deposited in NCBI Gene Expression Omnibus with the accession code PRJNA720297 and GSE171609. All data were expressed as mean ± standard error (SE).

### 2.9 Statistical analysis

All data in this study were presented as the mean ± SE. For blood analysis, gene expression data analysis, and metabolic analysis, differences between groups (Normal vs. HFD, HFD vs. HFD‐OVJ) were evaluated using unpaired Student's *t*‐tests. *p* < .05 was considered statistically significant.

## 3 RESULTS

### 3.1 Effects of organic vegetable juice on body weight, food efficiency ratio, epididymal fat and liver mass, and the size of adipocytes in diet‐induced obese mice

**FIGURE 2:**
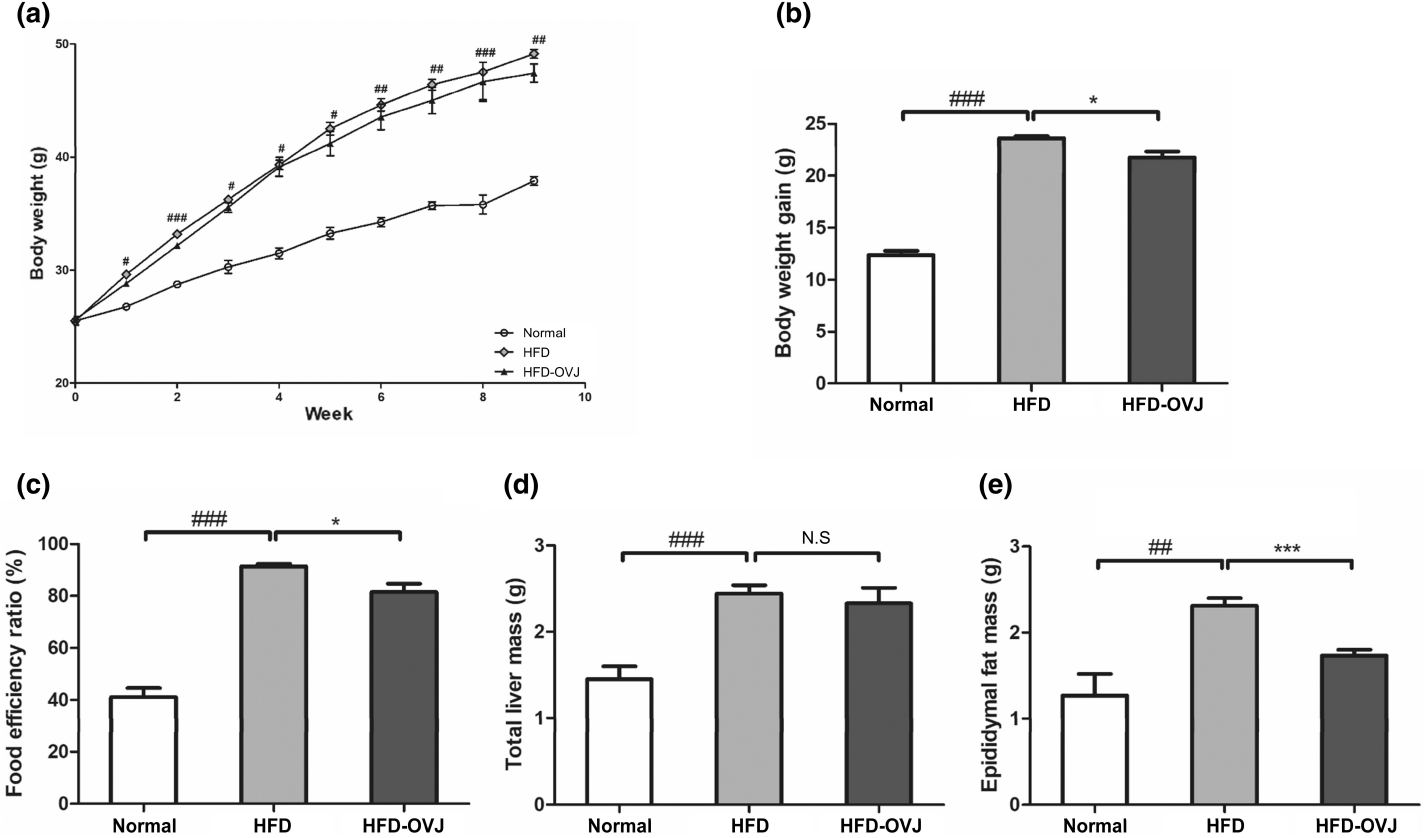
Effects of OVJ treatment on diet‐induced obese mice. (a) Change in body weight. (b) Body weight gain. (c) Food efficiency ratio. (d) Total liver mass. (e) Epididymal fat mas. Results are presented as the mean ± SE. Significant differences are indicated as ^#^ *p* < .05, ^##^ *p* < .01, and ^###^ *p* < .001 when compared with the normal group. *p < .05, **p < .01, ***p < .001 when compared with the HFD group. HFD, high‐fat diet; HFD‐OVJ, organic vegetable juice with high‐fat diet

Body weight of the HFD group was significantly increased after only 1 week of the HFD. Final body weight was 30% higher in the HFD group than in the normal group (*p* < .05). Body weight of the HFD‐OVJ group was slightly lower than that of the HFD group, but the difference was not significant (Figure 2a). After 9 weeks, the weight gain in the HFD group was significantly increased compared to the normal group (*p* < .001). Weight gain of mice in the HFD‐OVJ group was significantly lower than that of the HFD group (*p* < .05) (Figure 2b). The food efficiency ratio of the HFD group was significantly higher than that of the normal group (*p* < .001), but was significantly decreased when the HFD was supplemented with OVJ (*p* < .05) (Figure 2c).

The HFD group showed increased liver mass compared to normal group (*p* < .001). The liver mass of the HFD‐OVJ group was slightly lower than that of the HFD group, but there was no significant difference (Figure 2d). The epididymal fat mass of the HFD group was also increased by 78.5% compared to that in the normal group (*p* < .001). The weight of epididymal fat in the HFD‐OVJ group was significantly lower than that in the HFD group (*p* < .01) (Figure 2e).

**FIGURE 3:**
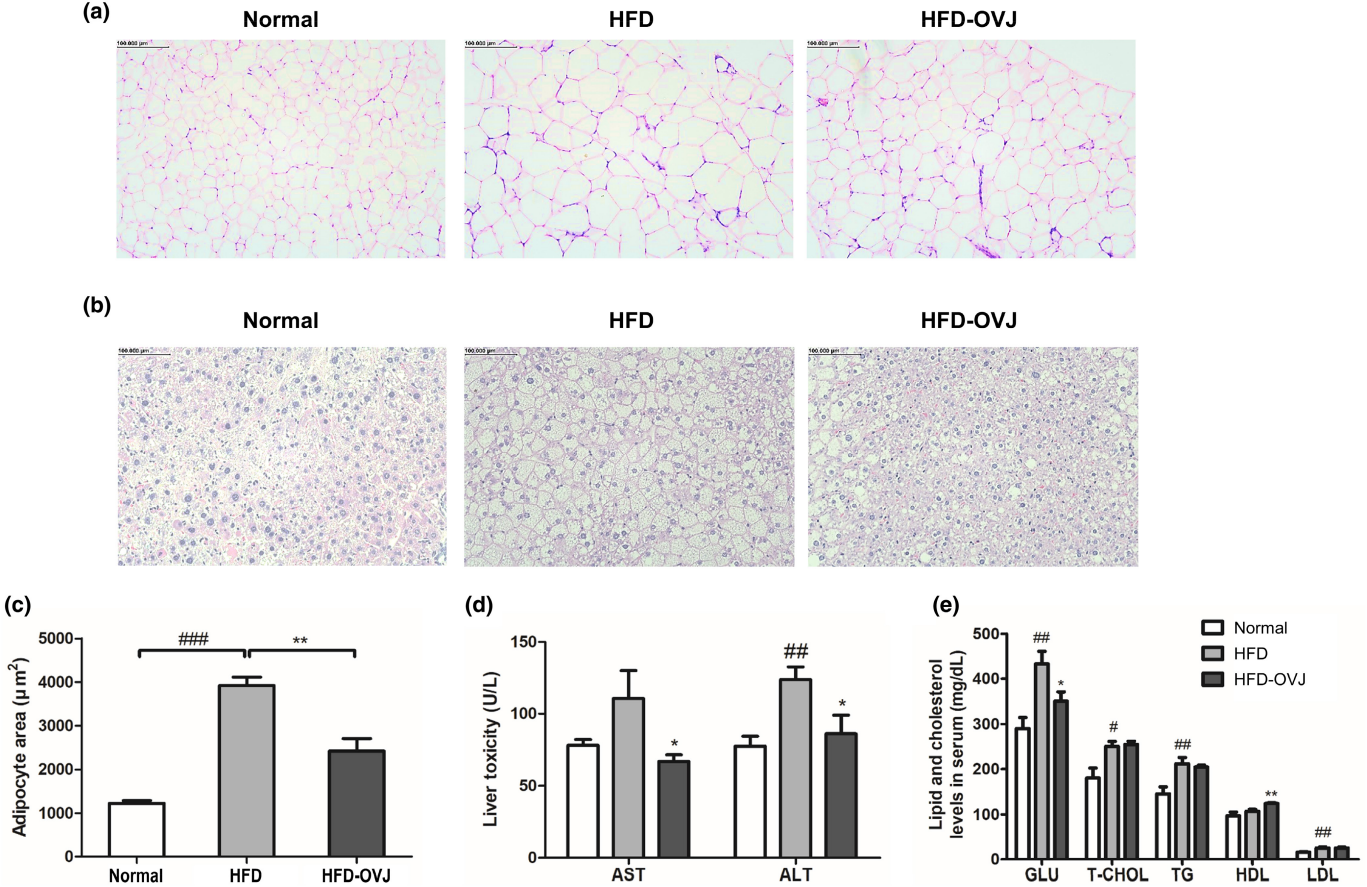
Effects of OVJ treatment on liver and epididymal fat of diet‐induced obese mice. (a) Morphology of epididymal fat, and (b) liver was analyzed using a microscope. (c) Adipocytes of epididymal fat. Effects of fruit‐vegetable drink on serum biochemistry and lipid and cholesterol levels of diet‐induced obese mice. (d) Serum biochemistry. (e) Lipid and cholesterol levels in serum. Results are presented as the mean ± SE. ^#^ *p* < .05, ^##^ *p* < .01, and ^###^ *p* < .001 when compared with the normal group, *p < .05 **p < .01 when compared with the HFD group. ALT, alanine transferase; AST, aspartate transferase; GLU, glucose; HDL, high‐density lipoprotein cholesterol; HFD, high‐fat diet; HFD‐OVJ, organic vegetable juice with high‐fat diet; LDL, low‐density lipoprotein cholesterol; T‐CHOL, Total cholesterol; TG, triglyceride

The adipocyte size in the liver and epididymal fat was measured using histological tissue analysis. The epididymal fat adipocyte was markedly enlarged in HFD group compared with the normal group. However, adipose tissue was reduced in the HFD‐OVJ group (Figure 3a). The degree of hepatic steatosis in the HFD group developed compared with the Normal group, but mice fed OVJ showed reduced steatosis compared to the HFD group (Figure 3b). The adipocyte area of epididymal fat showed significant reductions in the HFD‐OVJ group compared to the HFD group (*p* < .01) (Figure 3c). Intake of OVJ decreased epididymal fat mass and adipocyte formation in diet‐induced obese mice.

### 3.2 Effects of OVJ on serum biochemistry and lipid and cholesterol levels in diet‐induced obese mice

The levels of liver toxicity biomarkers AST and ALT were increased in the HFD group compared with the normal group, but only statistically significant for ALT (*p* < .01). AST and ALT levels were significantly reduced in the HFD‐OVJ group (*p* < .05) (Figure 3d). GLU levels were increased in the HFD group compared to the normal group (*p* < .01) and significantly decreased in the HFD‐OVJ group compared to the HFD group (*p* < .05). T‐CHOL and LDL were both increased in high‐fat diet‐induced obese mice (*p* < .05 and *p* < .01, respectively). The levels of T‐CHOL and LDL in the HFD‐OVJ group were similar to those in the HFD mice. A high‐fat diet increased TG levels in the serum to 211.83 ± 13.88 mg/dl compared with 145.43 ± 15.54 mg/dl in the normal group (*p* < .01). The level of TG in the HFD‐OVJ group (204.50 ± 4.19 mg/dl) was slightly lower than that in the HFD group, but the difference was not significant. The levels of HDL in the HFD and HFD‐OVJ groups were both higher than those in the normal group; especially, the HDL level of HFD‐OVJ fed mice was significantly increased compared to that of the HFD group (*p* < .01) (Figure 3e).

### 3.3 Effects of OVJ on lipid synthesis and inflammatory gene expression in the liver tissue of diet‐induced obese mice

**FIGURE 4:**
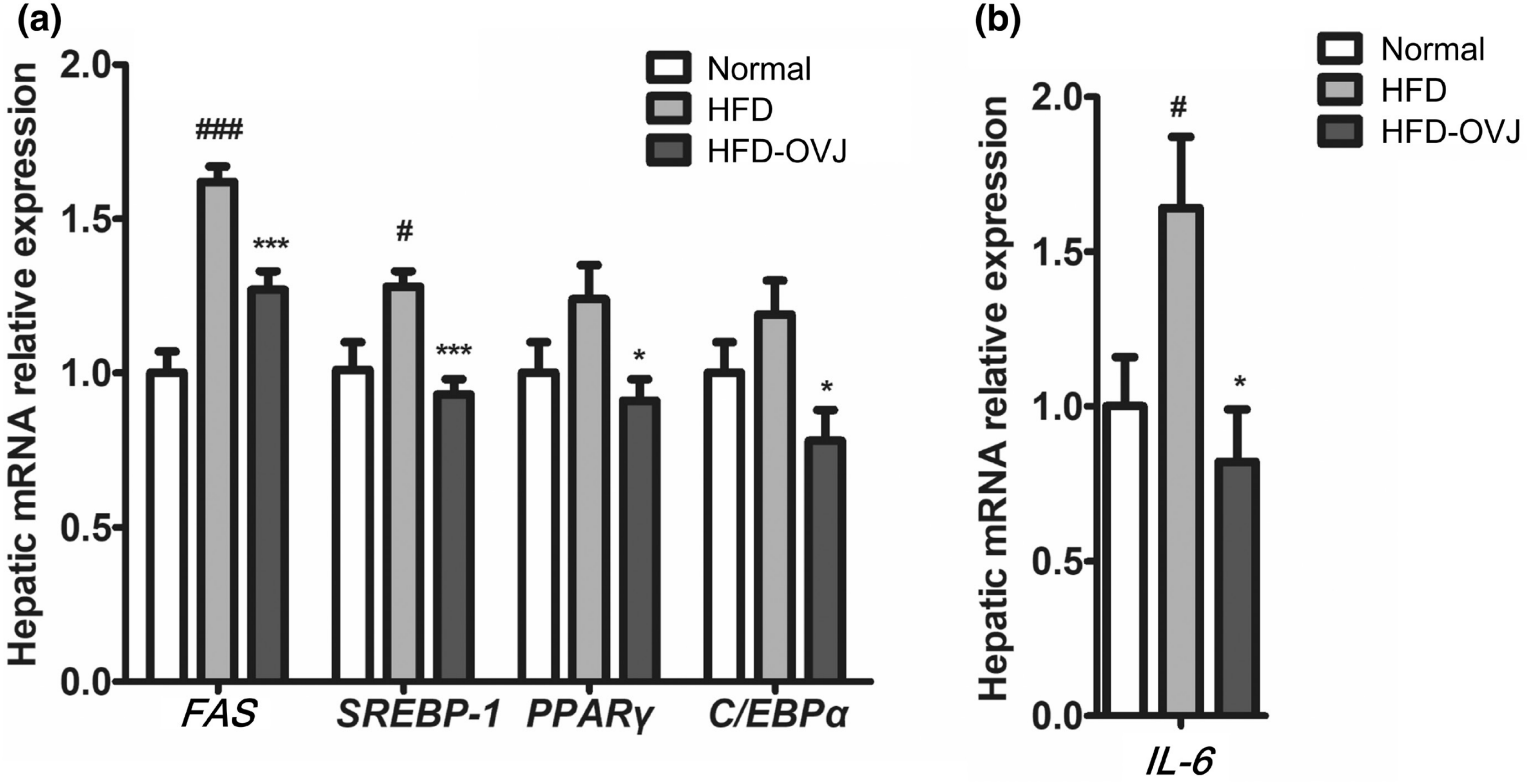
HFD and OVJ effect on the gene expression in the liver. (a) FAS, SREBP‐1, PPARγ, and C/EBPα, and (b) IL‐6. Results are presented as the mean ± SE. ^#^ *p* < .05 and ^###^ *p* < .001 when compared with the normal group, *p < .05 ***p < .001 when compared with the HFD group. HFD, high‐fat diet; HFD‐OVJ, organic vegetable juice with high‐fat diet

We examined gene expression related to lipid synthesis in liver tissues. The HFD group showed increased expression of genes involved in the regulation of sterol regulatory element‐binding protein 1 (SREBP‐1), peroxisome proliferator‐activated receptor (PPARγ), CCAAT/enhancer‐binding protein α (C/EBPα) and fatty acid synthesis (FAS) (Figure 4a). The expression of SREBP‐1 and FAS was significantly increased in HFD‐fed mice (*p* < .001 and *p* < .05, respectively). Lipid synthesis‐related gene expression, including FAS, SREBP1, PPARγ, and C/EBPα was significantly lower in the HFD‐OVJ group than in the HFD group (FAS and SREBP‐1, *p* < .001; PPARγ and C/EBPα, *p* < .05). We measured the mRNA levels of genes related to inflammation (Figure 4b). IL‐6 expression was significantly higher in the HFD group compared to that in the normal group (*p* < .05). In addition, HFD‐OVJ intake decreased the expression of SREBP‐1, PPARγ, and C/EBPα, and IL‐6 compared to HFD group.

### 3.4 Antioxidant effects of OVJ in diet‐induced obese mice

**FIGURE 5:**
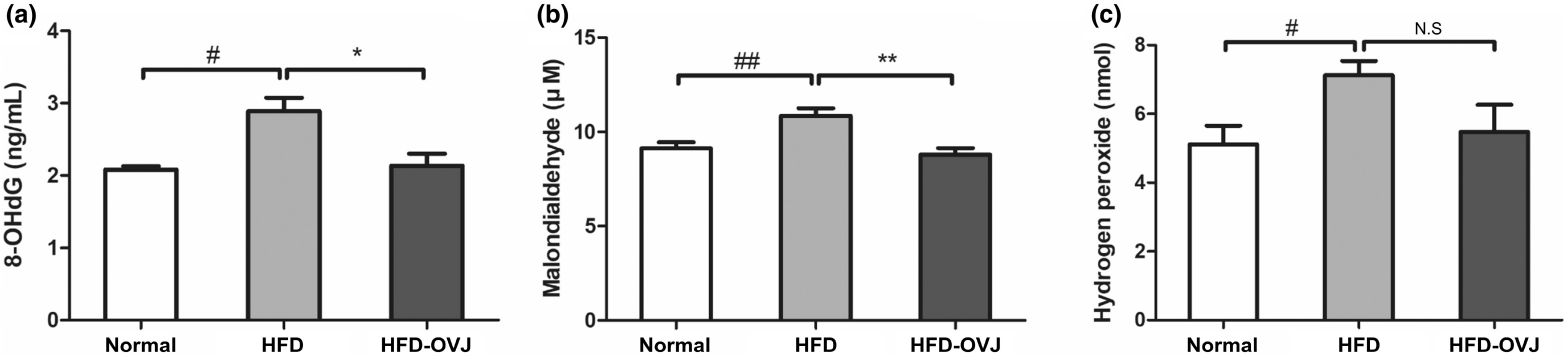
Antioxidant effects of OVJ. (a) 8‐OHdG, (b) MDA, and (c) H2O2 in diet‐induced obese mice. Results are presented as the mean ± SE. ^#^ *p* < .05 and ^##^ *p* < .01 when compared with the normal group, *p < .05, **p < .01 when compared with the HFD group. HFD, high‐fat diet; HFD‐OVJ, organic vegetable juice with high‐fat diet

8‐OHdG causes oxidative DNA damage via ROS. Therefore, 8‐OHdG is an established biomarker of oxidative stress. A high‐fat diet significantly increased 8‐OHdG in the serum compared with the normal group (*p* < .05). The level of 8‐OHdG in the HFD‐OVJ group was significantly lower than that in the HFD group (*p* < .05). In addition, the intake of OVJ in diet‐induced obese mice reduced 8‐OHdG activity to the level of the normal group (Figure 5a). MDA was used as a marker of lipid peroxidation by oxidative degradation of lipids. MDA concentration in the HFD group was significantly higher than that in the normal group (*p* < .01), while the MDA level in the HFD‐OVJ group was significantly lower than that in the HFD group (*p* < .01) (Figure 5b). H2O2 level was significantly elevated in the HFD group compared to the normal group (*p* < .05), but slightly reduced in the HFD‐OVJ group; there was no significant difference (Figure 5c).

### 3.5 Overview and diversity analysis of the microbial community in the culture system

**FIGURE 6:**
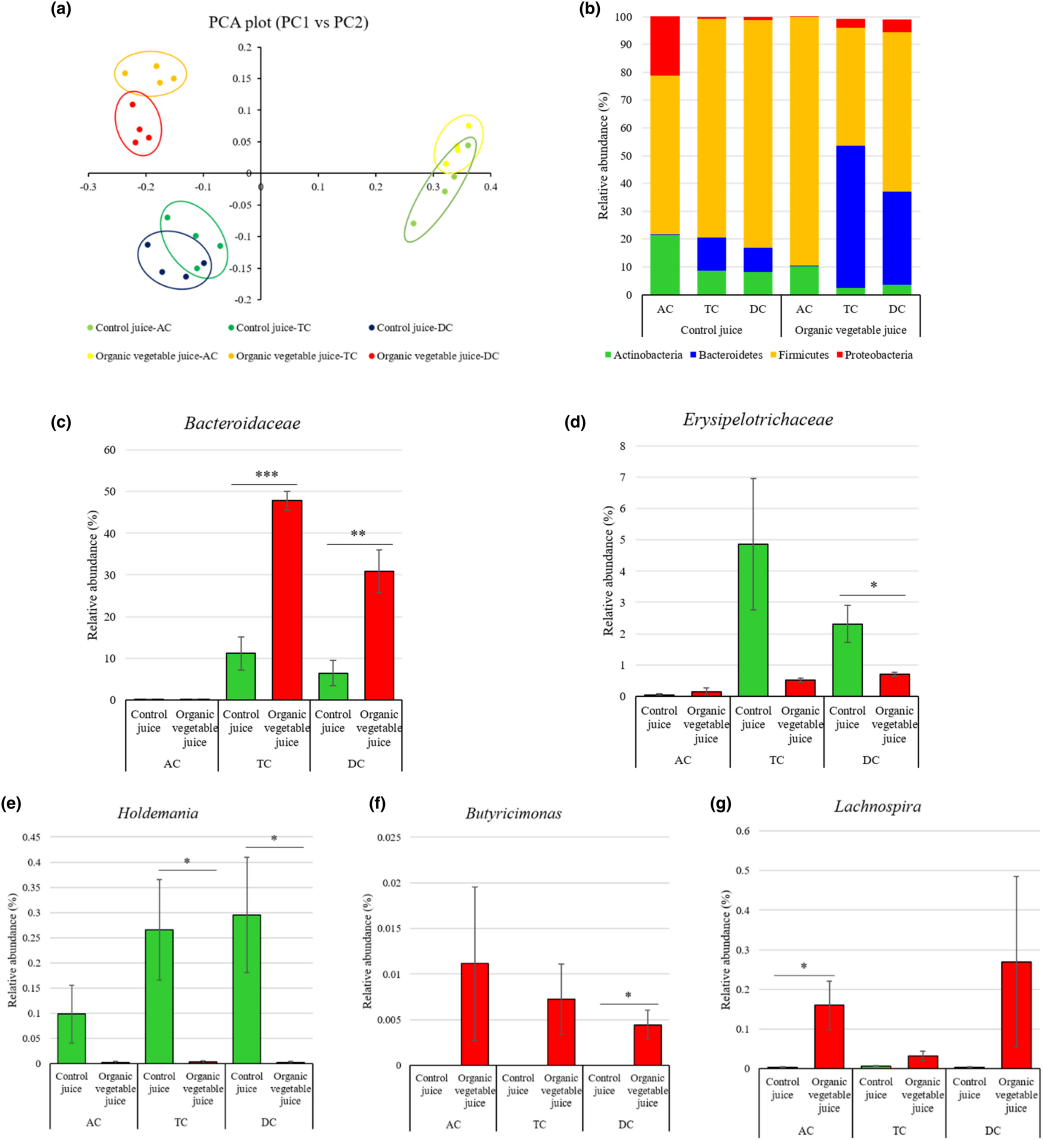
Effects of organic vegetable juice on the microbial composition and diversity of continuous colon simulation system. (a) PCA plot of beta‐diversity, (b) relative abundance of major phyla in each group. (c) Relative abundance of Bacteroidaceae. (d) Relative abundance of Erysipelotrichaceae. (e) Relative abundance of *Holdemania*. (f) Relative abundance of *Butyricimonas*. (g) Relative abundance of *Lachnospira*. *p < .05, **p < .01, and ***p < .001 when compared with the control juice. AC, ascending colon; DC, descending colon; TC, transverse colon

The microbial composition of the continuous colon simulation was analyzed using the Illumina MiSeq platform, targeting the variable region from V3 to V4 of the bacterial 16S rRNA gene with the 341F/805R primer set. After low‐quality sequence filtering, chimera checking, and OTU clustering, we confirmed an average of 190 OTUs with 97% sequence identity. We obtained an average of 35,512 high‐quality sequences per sample and classified reads were obtained. Alpha diversity, such as the Shannon diversity index, was used to measure the biodiversity and richness of all groups. Table 1 shows the alpha diversity of AC, TC, and DC for each control juice group and OVJ group. When control juice and organic juice treatment were processed, the average alpha‐diversity was identified as 2.66 and 2.79, respectively. These results indicated that the microbial community after OVJ treatment was slightly more diverse compared to that after control juice treatment. The Shannon index was significantly increased in the TC and DC after organic juice treatment (*p* < .01 and *p* < .05, respectively). After treatment with control juice, the Shannon diversity was also significantly increased in the TC, but there was no significant difference in the DC compared to the AC. The beta‐diversity showed by principal coordinates analysis, including classified OTUs, confirmed a difference in the microbial composition as a result of colon simulation parts after organic juice and control juice treatment (Figure 6a). When control juice and organic juice were added, the composition of the intestinal flora was changed according to different parts of the colon and sample type. The AC showed a similar beta‐diversity in the control juice and organic juice groups. Beta‐diversity of the transverse and descending parts showed alterations in the microbial community caused by control juice and organic juice treatment.

**TABLE 1:** Alpha diversity (Shannon index) of the bacterial community from continuous colon simulation system

Shannon index	Control juice				Organic vegetable juice			
	AC	TC	DC	AVG	AC	TC	DC	AVG
Stable	2.32 ± 0.37	2.80 ± 0.09	2.72 ± 0.15	2.67 ± 0.39	2.32 ± 0.37	2.80 ± 0.09	2.72 ± 0.15	2.56 ± 0.52
Treatment	1.75 ± 0.26	3.17 ± 0.04^##^	3.05 ± 0.04	2.66 ± 0.70	1.75 ± 0.17	3.38 ± 0.10^##^	3.25 ± 0.12^#^	2.79 ± 0.77
Washout	1.78 ± 0.04	3.02 ± 0.03	3.12 ± 0.38	2.64 ± 0.65	1.67 ± 0.31	2.80 ± 0.31	3.02 ± 0.36	2.49 ± 0.68

*Note*: Significant differences are indicated as ^#^ *p* < .05 and ^##^ *p* < .01 when compared with the Shannon index of Stable period.

Abbreviations: AC, ascending colon; AVG, average; DC, descending colon; TC, transverse colon.

### 3.6 Effects of OVJ on the microbial community in the culture system

The microbiota of the OVJ and control juice‐treated groups was analyzed at the phylum level (Figure S1A, Table S3). We analyzed four major phyla; Actinobacteria, Bacteroidetes, Firmicutes, and Proteobacteria. Bacteroidetes and Firmicutes occupied most of the microbial composition in each group (Figure 6b). Bacteroidetes were significantly increased in the TC and DC of OVJ group compared with the control juice group (*p* < .001 and *p* < .05, respectively). Firmicutes abundance was significantly lower in the TC and DC of the OVJ group than in those of the control juice treatment but significantly increased in the AC compared with the control juice treatment (*p* < .05). Proteobacteria abundance was reduced significantly in the AC of the OVJ‐treated group compared to the control juice group (*p* < .01).

At the family level, the top 30 species obtained from the colon simulation system were analyzed (Figure S1B). The abundance of Bacteroidaceae was significantly higher in TC and DC of OVJ group than in those of the control juice group (*p* < .001 and *p* < .01, respectively). Bacteroidaceae belongs to Bacteroidetes, and the abundance of these species was decreased in the intestines of obese people (Figure 6c). The abundance of this species was higher in the TC and DC of OVJ group than in those of the control juice group. In particular, there was a significant difference in DC (*p* < .05) (Figure 6d).

We compared changes in the relative abundance of the top 40 at the genus level (Figure S1C). The abundance of *Holdemania* was significantly higher in TC and DC of control juice group than in those of the OVJ‐treated group (*p* < .05) (Figure 6e). *Butyricimonas* occupied a very small percentage of the OVJ‐treated group, but this species did not exist in the control juice group. There was a significant difference in DC (*p* < .05) (Figure 6f). *Lachnospira* abundance was significantly increased in the AC of the OVJ group compared to the control juice group (*p* < .05) and was higher in the TC and DC of the control juice‐treated group (Figure 6g).

**FIGURE 7:**
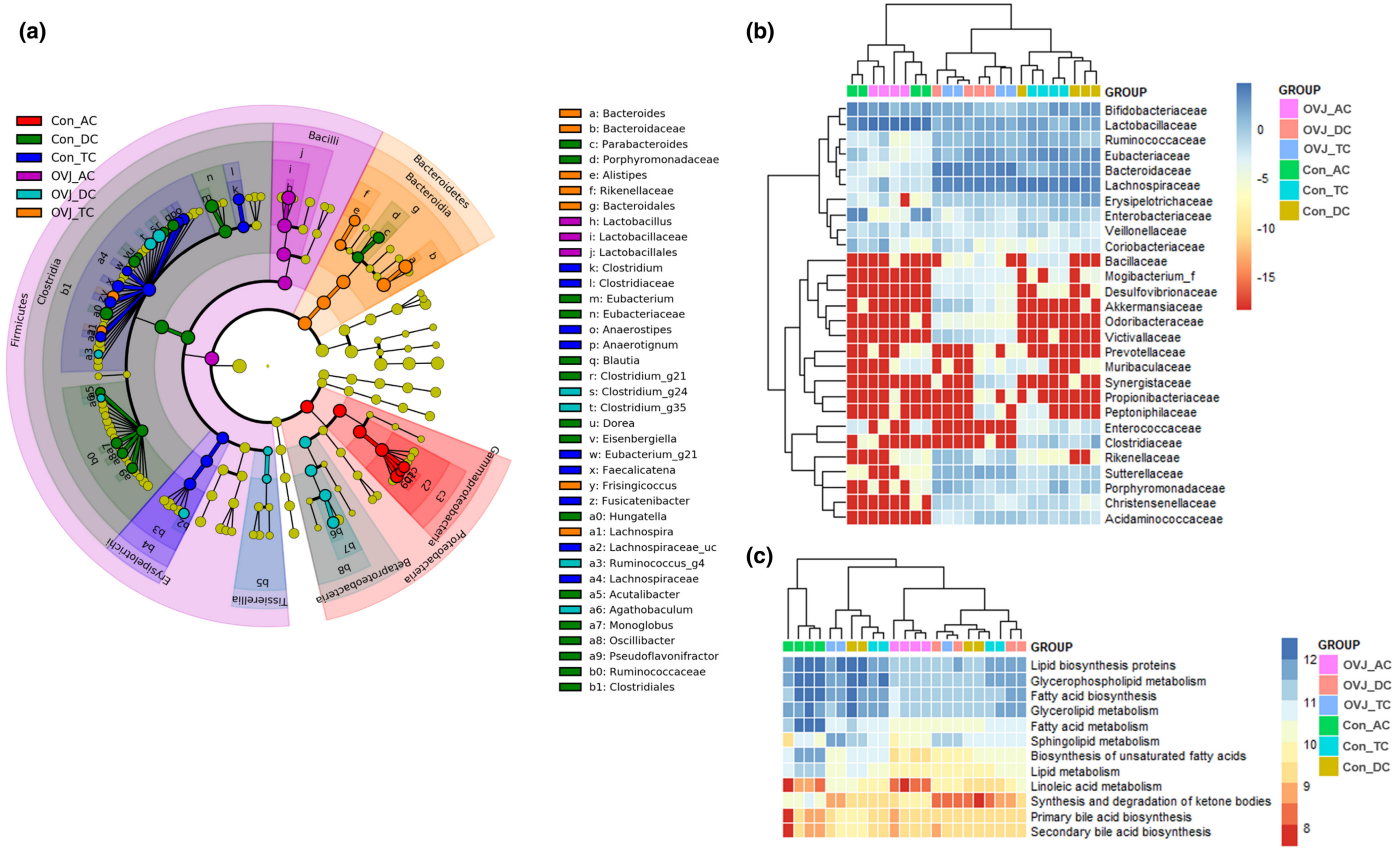
Effects of OVJ on the bacterial abundance and metabolic pathway of continuous colon simulation system. (a) Cladogram based on the relative abundance of microbial composition using LEfSe. (b) Heatmap showing the relative abundance of microorganism at the family level for each group. (c) Heatmap representing PICRUSt analysis with KEGG pathway of each group. AC, ascending colon; Con, control juice; DC, descending colon; OVJ, organic vegetable juice; TC, transverse colon

The LDA score was used to confirm the significant difference in the microbial composition in the colon section. There was a significant difference in microbial taxa level with a log LDA score above 3.0 for each colon section. In the AC of the OVJ treatment group, *Lactobacillus* abundance was significantly higher than in that of the other groups. Bacteroidales, *Frisingicoccus*, *Lachnospira*, and *Alistipes* abundance was significantly higher in the TC of the OVJ group than in that of the control group. *Clostridium*_g24, *Clostridium*_g35, *Ruminococcus*_g4, and *Agathobaculum* abundance were significantly increased in the DC of the OVJ group. *Escherichia* abundance was significantly higher in the AC of the control juice treatment group than in that of the OVJ group. *Fusicatenibacter*, *Faecalicatena*, and *Anaerostipes* abundance were significantly increased in the TC of the control juice group. In DC of control group, *Eubacterium*, *Hungatella*, *Elsenbergiella*, and *Blautia* abundance were significantly higher than in the other groups (Figure 7a).

At the family level, 28 bacterial genera showed differences among different groups. The abundance of Akkermansiaceae was increased in the TC and DC of the OVJ group compared to the control juice group (Figure 7b). We predicted the metagenome function of each taxonomy group using the Kyoto Encyclopedia of Genes and Genomes (KEGG) pathways. We investigated the predicted lipid metabolism function genes of the microbiome in OVJ and control juice groups with PICRUSt (Figure 7c). OVJ significantly changed the microbial functions, such as primary and secondary bile acid biosynthesis (LDA score: 4.13, 4.22, respectively). However, in the control juice group fatty acid biosynthesis (LDA score: 4.02), fatty acid metabolism (LDA score: 5.18), and lipid metabolism (LDA score: 4.83) were significantly shifted (Figure S2).

## 4 DISCUSSION

Obesity causes hyperlipidemia, which increases mortality by causing cardiovascular disease when the degree is severe. Therefore, it is necessary to lower the risk of cardiovascular disease by regulating blood lipids (Klop et al., 2013; Nelson, 2013). The human gut microbiota has been a major research topic in human health. A growing number of studies have suggested that the gastrointestinal microbiome is not only important for gut health but also for diseases such as obesity, diabetes, and atopy (Larsen et al., 2010; Penders et al., 2007; Zhao, 2013). Vegetables are a functional source that reduces the risk of diseases, such as cancer, cardiovascular disease, as well as aging (Liu, 2003). Major phytochemicals in the OVJ used in this study were β‐carotene and lycopene (β‐carotene; 113.1 ± 7.6, lycopene; 30.8 ± 0.9). β‐carotene and lycopene are carotenoids that have anti‐obesity properties (González‐Castejón & Rodriguez‐Casado, 2011). Here, we aimed to study the alleviating effect of hyperlipidemia effect of OVJ through in vivo experiment. Furthermore, we analyzed the gut microbiome using a continuous colon simulation system and investigated the correlation between the community and the metabolic pathways related to lipid synthesis predicted gene functions through OVJ consumption.

A HFD with OVJ slightly reduced body and liver weight gain and significantly decreased epididymal fat gain. Adipocytes of epididymal fat were also decreased, indicating that OVJ supplement can reduce adipocyte size in the epididymal fat of obese mice (Duwaerts et al., 2017). Serum lipid and cholesterol levels of diet‐induced obese mice tended to increase, which are correlated with hyperlipidemia (Neyrinck et al., 2013; Zhang et al., 2007). OVJ decreased GLU and TG levels and increased blood HDL. When GLU concentration increases, insulin ratio increases, and the excess glucose is stored in the subcutaneous fat, causing hyperlipidemia (Koopmans et al., 2001). According to previous studies, fruit and vegetable juice may contribute to improving blood lipid profiles and further prevent cardiovascular diseases, including hyperlipidemia (Chang & Liu, 2009; Zheng et al., 2017). In other words, the results of this study suggested that the juice used in the study might also improve blood lipids. Lipid synthesis‐related gene expression was significantly decreased in the liver tissue of HFD‐OVJ mice, including SREBP‐1, PPARγ, C/EBPα, and FAS, which suggested lower lipid levels and lower fatty acid synthesis in OVJ‐treated mice (Hu et al., 2010; Park et al., 2013). FAS is a key factor in determining the maximum capacity to synthesize fatty acids via the de novo pathway (Clarke, 1993). PPARγ is a transcription factor that is mainly expressed in the adipose tissue and regulates the accumulation of fat in adipocytes by being involved in the production of insulin‐sensitive adipokines, such as adiponectin. It is also involved in the synthesis of TG (Medina‐Gomez et al., 2007; Park et al., 2019). SREBP‐1 is a transcription factor that plays an important role in the synthesis of TG in adipose and liver tissues. The expression of SREBP‐1 is dominant in liver tissues, and it regulates the expression of enzymes related to TG synthesis in hepatocytes (Shimano et al., 1999; Yuan et al., 2009). Hyperlipidemia is accompanied by an increase in free fatty acids in the blood. Increased blood‐free fatty acids are directly toxic to hepatocytes (Feingold & Grunfeld, 1992). The increase in free fatty acids in the liver increases the activity of enzymes that generate free radicals, lipid peroxidation, and the production of inflammatory cytokines such as IL‐6. Oxidative stress is associated with obesity‐related complications, such as hyperlipidemia. Production of 8‐OHdG is caused by oxidative DNA damage, which increases in overweight and obese people. The level of 8‐OHdG in obese women is significantly higher than that in lean women (Devries et al., 2008). MDA is the main product of lipid peroxidation, which is a free radical‐generating process by oxidants (Garcia‐Sanchez et al., 2020). The concentration of MDA was significantly reduced in patients with healthy BMI compared to that in obese individuals (BMI above 40 kg/m^2^) (Olusi, 2002). In this study, treatment with OVJ significantly decreased the lipid synthesis‐related genes and oxidative stress. It could be the effect of β‐carotene and lycopene. The anti‐obesity effect of β‐carotene is related to the provitamin A effect. This effect is associated with reduced expression of PPARγ in the adipose tissue through the involvement of retinol X receptor signaling (Mounien et al., 2019). Lycopene blocks lipid accumulation in the adipose tissue by decreasing the expression of lipogenesis‐related genes, which is also related to the reduction of inflammatory cytokines (Fenni et al., 2017; Wang et al., 2019). Given this, it is possible to predict vegetables influenced improvement of blood lipids and antioxidant activity in animal experiments.

We examined the positive alterations of the microbiome and metagenomic functions in each section of the continuous colon simulation system. OVJ treatment significantly improved the richness of microorganisms in TC and DC and caused distinct alterations in the composition of the gut microbiome. The relative abundance of the family Erysipelotrichaceae and the genus *Holdemania* was significantly reduced in the DC of OVJ‐treated group compared to the control group. Previous studies have reported that Erysipelotrichaceae exhibits high abundance in obese individuals (Zhang et al., 2009), and that there is a correlation between Erysipelotrichaceae levels and host cholesterol metabolites (Martínez et al., 2013). In addition, it has been reported that supplementation of flavonol quercetin inhibits the growth of Erysipelotrichaceae (Etxeberria et al., 2015). The genus *Holdemania* has been reported to correlate with clinical markers of impaired lipid and glucose metabolism (Lippert et al., 2017). Our study shows that supplementation of OVJ can inhibit the growth of taxa associated with obesity and lipid metabolism, such as Erysipelotrichaceae and *Holdemania*. The three taxa whose relative abundance were increased significantly in the OVJ‐treated group compared to the control group were the family Bacteroidaceae in TC and DC, the genus *Butyricimonas* in DC and the genus *Lachnospira* in AC. Bacteroidaceae family is known to be significantly decreased in obesity, and the genus *Bacteroides* spp. has been reported to indicate a negative correlation between energy intake and obesity (Chávez‐Carbajal et al., 2019). *Butyricimonas* is known as butyrate‐producing bacteria with anti‐inflammatory effects (Den Besten et al., 2013). *Lachnospira* also belongs to butyrate‐producing bacteria and is known for the fermentation of polysaccharides of SCFAs (Ferrario et al., 2014). Butyrate has very beneficial effects on energy metabolism, intestinal homeostasis, and regulation of immune response, and has the potential to alleviate obesity and related comorbidities by regulating liver and intestinal lipid metabolism (Coppola et al., 2021). In addition, OVJ consumption increases the relative abundance of *Lactobacillus* and *Akkermansia*, known as beneficial bacteria. According to Wiese et al. lycopene‐rich food and flavonol consumption increase the relative abundance of *Lactobacillus* and change liver metabolism and vascular functions (Wiese et al., 2019). Previous research supports the link between the gut and vascular systems, which also links risk factor‐mediated cardiovascular diseases (Li et al., 2017). According to Chang et al., *Akkermansia*, belonging to the Akkermansiaceae family, is related to reduced weight gain and maintenance of metabolic homeostasis (Baldwin et al., 2016) and is enriched in healthy people rather than people with metabolic syndrome (Lim et al., 2017). We also observed that the OVJ‐treated group and control juice‐treated group showed a shift in metagenome function, related to the lipid metabolism. KEGG pathway analysis showed a lower number of genes related to lipid metabolism in OVJ‐induced vessels. This suggested that the administration of OVJ improved the microbiome environment and potentially reduced lipid metabolism‐related gene expression. In our results, treatment with OVJ increased the abundance of butyrate‐producing bacteria and therefore OVJ treatment could increase endogenous butyrate production and could be a useful strategy for the prevention of obesity and related metabolic diseases.

In this study, we showed that the microbial community and lipid metabolism were altered in the culture system upon treatment with OVJ, and that blood lipid profiles and antioxidant ability were alleviated in diet‐induced obese mice. These results suggest that OVJ may represent a natural way of alleviating hyperlipidemia. In future studies, it may be necessary to determine whether OVJ influences lipid metabolism‐related genes directly or whether its effects are predominantly mediated by changes in the microbiome. In addition, it may be necessary to investigate the effects of the ingredients on the lipid metabolic gene pathways of microbiome to improve blood lipid profiles and hyperlipidemia in future studies.

## FUNDING INFORMATION

This research received no external funding.

## CONFLICT OF INTEREST

The authors declare no conflict of interest.

## Supporting information


Table S1.
Table S2.
Table S3.
Figure S1.
Figure S2.

## Data Availability

Data are contained within the article or supplementary material.
