# Supplementary material for: Organic vegetable juice supplement alleviates hyperlipidemia in diet‐induced obese mice and modulates microbial community in continuous colon simulation system
Source: Food Sci Nutr. 2023 Jan 13;11(3):1531–43. doi: 10.1002/fsn3.3193 (PMC10002948; doi:10.1002/fsn3.3193)
Supplement: Supplementary file 1 — Table S1. Table S2. Table S3. Figure S1. Figure S2. [file FSN3-11-1531-s001.docx]

Table S1: The composition of Organic Vegetable Juice, Table S2: Gene names and symbols used in gene expression analysis, Figure S1: Changes in microbial composition according to timing and colon vessels after sample treatment at (A) phylum level, (B) family level, (C) genus level, Table S3: Changes in microbial composition of colon vessels after sample treatment at phylum level, Figure S2: Histogram of the linear discriminant analysis (LDA) score of predicted gene function according to the colon section.
